# Temsirolimus Partially Rescues the Hutchinson-Gilford Progeria Cellular Phenotype

**DOI:** 10.1371/journal.pone.0168988

**Published:** 2016-12-29

**Authors:** Diana Gabriel, Leslie B. Gordon, Karima Djabali

**Affiliations:** 1 Epigenetics of Aging, Department of Dermatology, TUM school of Medicine, Technical University of Munich (TUM), Garching-Munich, Germany; 2 Department of Pediatrics, Hasbro Children’s Hospital, Warren Alpert Medical School of Brown University, Providence, Rhode Island, United States of America; 3 Department of Anesthesia, Boston Children’s Hospital, Harvard Medical School, Boston, Massachusetts, United States of America; Univerzitet u Beogradu, SERBIA

## Abstract

Hutchinson-Gilford syndrome (HGPS, OMIM 176670, a rare premature aging disorder that leads to death at an average age of 14.7 years due to myocardial infarction or stroke, is caused by mutations in the *LMNA* gene. Lamins help maintain the shape and stability of the nuclear envelope in addition to regulating DNA replication, DNA transcription, proliferation and differentiation. The *LMNA* mutation results in the deletion of 50 amino acids from the carboxy-terminal region of prelamin A, producing the truncated, farnesylated protein progerin. The accumulation of progerin in HGPS nuclei causes numerous morphological and functional changes that lead to premature cellular senescence. Attempts to reverse this HGPS phenotype have identified rapamycin, an inhibitor of mammalian target of rapamycin (mTOR), as a drug that is able to rescue the HGPS cellular phenotype by promoting autophagy and reducing progerin accumulation. Rapamycin is an obvious candidate for the treatment of HGPS disease but is difficult to utilize clinically. To further assess rapamycin’s efficacy with regard to proteostasis, mitochondrial function and the degree of DNA damage, we tested temsirolimus, a rapamycin analog with a more favorable pharmacokinetic profile than rapamycin. We report that temsirolimus decreases progerin levels, increases proliferation, reduces misshapen nuclei, and partially ameliorates DNA damage, but does not improve proteasome activity or mitochondrial dysfunction. Our findings suggest that future therapeutic strategies should identify new drug combinations and treatment regimens that target all the dysfunctional hallmarks that characterize HGPS cells.

## Introduction

Hutchinson-Gilford progeria syndrome (HGPS, OMIM 176670) is a rare genetic disorder that occurs in 1 to 4 million live births [1]. The genetic basis of most HGPS cases is the G608G mutation (GGC>GGT) occurring within exon 11 of the lamin A gene [2], which results in the activation of a novel cryptic splice [2, 3]. The protein produced, progerin, lacks 50 amino acids near the prelamin A carboxyl terminus and remains permanently farnesylated [2, 3]. The consequent persistent anchoring of progerin to the nuclear envelope disrupts the nuclear lamina and causes nuclear blebbing, disorganized heterochromatin and DNA double-strand break accumulation in both human HGPS cells and mouse transgenic cells [1, 4–10]. Farnesyltransferase inhibitors (FTIs) have thus far proven to be effective for HGPS [1]. Indeed progerin-induced defects in the nucleus were reduced after FTI treatment [11–16]. The results of the first clinical drug trial using an FTI (Ionarfarnib) are promising, as children demonstrated weight gain, improved bone structure, increased blood vessel flexibility [1], and slightly improved estimated lifespan [17]. Nevertheless, FTIs appear to induce disruption of the lamin B network, the formation of donut-shaped nuclei, and increased DNA double-strand breaks in HGPS cells [7, 18–20]. Therefore, the identification of new compounds that could promote the degradation of progerin will be of great interest for correcting the HGPS cellular phenotype.

The macrocyclic antibiotic rapamycin has long been used as an antifungal and immunosuppressive agent and, more recently, it has been shown to improve aging diseases, such as neurodegenerative disorders and arteriosclerosis [21]. Its major cellular target, mammalian target of rapamycin (mTOR), regulates cell growth, hormonal signals, and cell proliferation; when inhibited mTOR, activates autophagy [22]. Although rapamycin was shown to extend lifespan in a number of species, including mice and flies, even when treatment was initiated late in life [21, 23], only reduced effects on mammalian aging rates have been reported [24]. In fact, rapamycin might have benefits without exerting a direct effect on aging, as a number of age-related diseases were improved by rapamycin, such as cancer incidence, immune deficiency, myocardial pathology, and arterial degeneration, whereas others were not measurably altered [24]. Critical evaluation of these findings suggests that rapamycin promotes longevity by targeting some core molecular processes that drive cellular and systemic aging, but not all [25].

Treating HGPS fibroblasts with rapamycin results in decreased progerin levels, attributed to increased progerin clearance via autophagy activation, which consequently leads to amelioration of the HGPS cellular phenotype [26]. The beneficial effect on HGPS fibroblast longevity supports rapamycin treatment as a potential therapeutic avenue for HGPS children. Nevertheless, the side effects of rapamycin, which include gastrointestinal symptoms, delayed wound healing, and interstitial pneumonitis, should be taken into account [27, 28].

We have therefore tested a soluble ester of rapamycin that exhibits fewer side effects when administered clinically; the analog temsirolimus [29, 30]. To determine its effect on proteostasis, mitochondrial function, DNA damage, proliferation and progerin levels, in this study, we investigated the effects of short-term and long-term temsirolimus treatments on HGPS primary fibroblast cultures compared with normal fibroblasts.

## Materials and Methods

### Cell culture and drug treatments

Fibroblast cultures were obtained from The Progeria Research Foundation Cell and Tissue Bank (www.progeriaresearch.org) were derived from HGPS patients: HGADFN003, HGADFN127, HGADFN155, HGADFN164, and HGADFN188. Control fibroblast cultures were obtained from the Coriell Institute for Medical Research (Camden, NJ, USA): GM01651C, GM0323B, GM03349C, GM03348E, and GM08398A. Cells were cultured at 37°C and 5% CO_2_ in DMEM high glucose medium (4.5 g/L glucose and sodium bicarbonate, without L-glutamine and sodium pyruvate) containing 15% FBS, 1% glutamine, 1% PenStrep, and 0.5% gentamicin. All experiments described in this study were performed using at least 3 controls and 3 HGPS primary fibroblast lines between passages 10 to 16 that had been cultured in parallel to ensure the same culture conditions. All described conditions for one experiment were investigated simultaneously to compare of HGPS cells to control. Negative and positive controls were added to assays: tamoxifen (10 μM for 24h at 37°C, positive control), trypsin (1h at 37°C, positive control), TBHP (50 μM for 2-3h at 37°C, positive control), pycocyanin (300 μM for 10 min at 37°C, positive control), N-acetyl-L-cysteine (5 mM for 30 min at 37°C, negative control), antimycin A for oxygen consumption (undiluted, negative control), for glycolysis (undiluted, positive control), and for mitotoxicity (undiluted, positive control).

Temsirolimus (Sigma-Aldrich) was added to the medium at a concentration of 1.0 μM. The medium was changed daily for the indicated periods of time. In parallel, mock-treated fibroblasts were cultured with medium containing vehicle (DMSO). Proteasome activity was blocked by 1.0 μM MG132 (Sigma-Aldrich) for 12 h; 25 μM chloroquine (Sigma-Aldrich) was applied for 12 h to block lysosomal activity in the presence or absence of 1.0 μM temsirolimus.

Everolimus (Sigma-Aldrich) was tested by comparison to temsirolimus and used at a concentration of 1.0 μM.

To analysis of the mitochondrial activity, DMEM high glucose medium was replaced with galactose-containing medium. Galactose medium was supplemented with 15% FBS, 10 mM galactose, 1.0 mM sodium pyruvate, 1% glutamine, 1% PenStrep, and 0.5% gentamicin.

### Cell toxicity

Cell toxicity was determined using a Cell Tox Green Kit (Promega, Mannheim, Germany) according to the manufacturer’s instructions. Higher concentrations of Temsirolimus resulted in higher cell death. Given this, a concentration of 1.0 μM temsirolimus was selected for all experiments.

### Cumulative population doubling determination

Control and HGPS cells were seeded in triplicate at a known density per 10cm dish and cultured in DMEM high glucose medium at 37°C for the indicated periods of time. Afterwards, the cells were harvested, and the number of cells was measured with a CASY Cell Counter (Roche, Penzberg, Germany). The numbers of cumulative population doublings (CPDs) were determined using the following formula:

n=3.32 (log Xh ‒ log Xb)+S

n is the total number of CPDs, Xh is the number of cells seeded. Xs is the number of cells harvested. S is the starting CPD as described [31].

### Western blot analysis

Cell pellets were resuspended in Laemmli sample buffer (BioRad), and Western blots were performed as described previously [32]. The membranes were incubated with the following primary antibodies: anti-lamin A/C (kindly provided by Dr. N. Chaudhary (1:10000)) [33], anti-progerin (rabbit monoclonal, 0.1 μg/mL) [34], anti-proteasome S20 subunit C2 (ab22665, Abcam, 1/4000), anti-ubiquitin (sc-8017, Santa Cruz Biotechnology, 1:3000), anti-LC3B (Sigma-Aldrich, 1:10000), anti-SQSTM1/p62 (ab56416, Abcam,1:2000), anti-53BP1 (A300-272A, Bethyl, 1:3000), anti-Rad51 (NBP2-32622, Novus Biological, 1:1000), anti-Nox4 (Novus Biological, 1:1000), anti-CoxII (Abcam, 1:1000), anti-Hsp27 (Abcam, 1:3000), anti-Ubiquitin (Santa Cruz Biotechnology, 1:3000), anti-pS6RP (4856, Cell Signaling, 1:1000), p4EBP1 (2855, Cell Signaling, 1:1000), anti-S6RP (Cell signaling, 1:1000), anti-4EBP1 (Cell signaling, 1:1000), and anti-β-actin (Sigma-Aldrich, 1:10000). Membranes were washed and incubated with a corresponding secondary antibody coupled to horseradish peroxidase (Jackson ImmunoResearch Laboratories). Proteins were visualized with a chemiluminescence detection system (ECL substrate; BioRad), and signals were analyzed using IMAGE LAB software (BioRad). Protein signals were quantified by normalizing to β-actin as indicated.

### Immunocytochemistry

For immunocytochemistry, fibroblasts were grown on coverslips, fixed with 100% methanol at -20°C for 10 min and were further processed for immunohistochemistry as previously described [32]. The following primary antibodies were used: anti-progerin S9 (1 μg/mL) [34], anti-lamin A/C (Chaudhary N, 1:500), anti-lamin B1 (sc-6217, Santa Cruz Biotechnology, 1:75), anti-γH2AX (JBW301, Millipore, 1:200), anti-53BP1 (A300-272A, Bethyl, 1:1000), anti-Rad51 (NBP2-32622, Novus Biological, 1:100), anti-Nox4 (Novus Biological, 1:1000), and anti-CoxII (Abcam, 1:1000). The secondary antibodies used were affinity-purified Alexa Fluor 488 goat or donkey IgG antibodies (Molecular Probes) and Cy3-conjugated IgG antibodies (Jackson ImmunoResearch). DAPI in Vectashield mounting medium (Vector Inc.) was used for counterstaining. The images were acquired (exposure time-matched) using an Axioplan fluorescence microscope (Carl Zeiss).

### Proteasome activity measurements

Treated and mock-treated fibroblasts were harvested and then counted using CASY cell counting technology (Roche Innovatis AG). The proteasome activity assay was performed according to the manufacturer’s instructions (Cayman 20S Proteasome Assay Kit, Cayman Chemical Company). Briefly, cell numbers were adjusted to 2.6 x 10^5^ and equal amounts were seeded in triplicate in a 96-well plate and allowed to attach overnight. After centrifugation, the cells were washed with 100 μL of assay buffer, followed by incubation with 100 μL of lysis buffer for 30 min. Then, samples were centrifuged for 10 min at 500 x g, and 90 μL of supernatant from each sample was then added to a new well in a plate; 10 μL of SUC-LLVY-AMC substrate was added and incubated at 37°C for 1 h. The fluorescence intensity at 360 and 480 nm was measured, and the 360/480 absorbance ratio was measured.

### Autophagy measurements

The numbers of autophagic vacuoles in treated and mock-treated fibroblasts were determined using an Autophagy/Cytotoxicity Dual Staining Kit (Cayman Chemical Company). Mock-treated and temsirolimus-treated cells were harvested, and equal amounts of cells were seeded in triplicate in a 96-well plate. The cells were allowed to attach overnight. The plate was centrifuged, and 100 μL of monodansylcadaverine (MDC) was added to the wells at a 1:1000 ratio. After incubation at 37°C for 10 min, the plate was centrifuged and washed twice with assay buffer. Autophagic vacuole intensities were measured at an excitation wavelength of 335 nm and an emission wavelength of 512 nm. Tamoxifen was used as a positive control as it induces the formation of autophagosomes without inducing cell death.

### Measurement of ROS

ROS in fibroblasts were measured using 2’,7’-dichlorofluorescein diacetate (DCFDA) with a Cellular ROS Detection Assay Kit from Abcam. Mock-treated and temsirolimus-treated cells were seeded at the same ratios in a 96-well plate and allowed to attach overnight. Adherent cells were incubated with 25 μL of DCFDA for 45 min at 37°C, and followed by washing. Fluorescence was measured using a POLARstar OMEGA (BMG Labtech).

### Measurements of ATP

Treated and mock-treated fibroblasts were assessed using a CellTiter-Glo Luminescent Cell Viability Assay (Promega) to determine the intracellular ATP content. Cells were harvested and equally seeded in triplicate in a 96-well plate. Then, cells were incubated with 100 μL of CellTiter-Glo reagent (CellTiter-Glo buffer plus CellTiter-Glo substrate) for 10 min, and the luminescence intensity was measured.

### Measurement of mitochondrial toxicity in fibroblasts

Fibroblasts were cultured in either glucose or galactose medium supplemented with 1.0 μM Temsirolimus. For assays, the cells were treated for 2 h with galactose ± Temsirolimus and compared to cells mock-treated with glucose. For comparison, cells cultured in glucose were treated with temsirolimus. The fibroblasts were examined with a Mitochondrial ToxGlo Assay from Promega according to the manual’s instructions.

Briefly, cells were harvested and equally seeded in a 96-well plate and were allowed to attach overnight at 37°C; then cells were treated with temsirolimus or vehicle for 2 hours or as indicated. For cytotoxicity evaluation, cells were incubated with the reagent for 30 min at 37°C before fluorescence was measured. The same plate was used to measure the intracellular ATP content, whereby the substrate was added to the wells and mixed for 5 min, followed by luminescence measurement. Antimycin A served as a positive control because it is cytotoxic and reduces ATP levels.

### Measurement of total ROS and superoxide

Detection of total ROS/reactive nitrogen species and the determination of superoxide production in living cells were performed using fluorescence microscopy and a microplate assay according to the manufacturer’s instructions.

For fluorescence microscopy, cells were grown on coverslips and treated with normal medium, temsirolimus or the vehicle. On the day of the experiment, the medium was replaced with fresh medium. For DNA staining, Hoechst33342 solution was added at a dilution of 1:10 for 1 hour at 37°C prior to antibody staining. A ROS inhibitor (N-acetyl-L-cysteine) was added 30 min before induction. For induction, the same volume of ROS/superoxide detection solution was added to the coverslips and incubated for 30 min at 37°C. After 20 min, ROS production was induced with pycocyanin, and the cells were incubated for another 10 min at 37°C. The coverslips were washed and covered with 1x washing buffer. Fluorescence was detected at Ex/Em: 490/525 nm and Ex/Em: 550/620 nm.

For fluorescence microplate assays, cells were cultured in normal medium and medium containing the vehicle or test compound. The day before the experiment, the cells were seeded equally in a 96-well clear-bottom/black-wall plate and allowed to attach overnight at 37°C. The cells were washed and treated according to the manufacturer’s instructions for the negative and positive controls. A 100 μl aliquot of ROS/superoxide detection solution was added and incubated for 60 min at 37°C. The plate was evaluated (bottom reading) with FLUOStar and standard fluorescein (488/520 nm) and rhodamine (550/610 nm) filter sets.

### Measurement of oxygen consumption and glycolysis

Cells were treated with either vehicle or temsirolimus and examined with MitoXpress Solution for oxygen consumption rates and lactate dehydrogenase for glycolysis levels. Briefly, cells were seeded in equal amounts in a 96-well clear-bottom/black-wall plate and allowed to attach overnight at 37°C. Then, 10 μl of MitoXpress Solution was added to each well, which was overlaid with 100 μl of HS Mineral Oil. The fluorescence intensity was measured at Ex/Em 380/650 nm at 37°C. Afterwards, glycolysis levels were measured by transferring 10 μl of each sample to a new clear 96-well plate: 90 μl of assay buffer and 100 μl of reaction solution were added to each well. After 30 minutes of gentle agitation, the absorbance was measured at 490 nm. Antimycin A was added as positive/negative control as it reduces oxygen consumption and increases glycolysis.

### Viability assay

Control and HGPS cells that were mock-treated or temsirolimus-treated were analyzed using a Muse Count and Viability Kit (Millipore) at the indicated time points. Briefly, cells were washed with PBS before trypsin was added to detach the cells. Then, cells were collected and centrifuged, and the supernatant was discarded. Cells were stained with the Muse Count and Viability reagent for 5 minutes at room temperature. By using a positive control (trypsin) that induces cell death, the Muse instrument settings were adjusted before control and HGPS fibroblasts were measured.

### Senescence detection via β-galactosidase staining

Control and HGPS fibroblasts were treated every other day with the vehicle or 1 μM temsirolimus for 55 days. Then, cells were split and grown on coverslips, washed once with PBS, fixed with 2% formaldehyde/0.2% glutaraldehyde for 5 minutes, and a staining solution composed of 1 mg/mL 5-bromo-4-chloro-3-inolyl-β-galactosidase in dimethylformamide (20 mg/mL stock), 5 mM potassium ferricyanide, 150 mM NaCl, 40 mM citric acid/sodium phosphate, and 2 mM MgCl2 at pH 6.0 was added (overnight at 37°C) [35]. The cells were then washed twice with PBS. DNA was stained with DAPI.

### Statistical analyses

Results are presented as the mean ± SD for all experiments. Comparisons were performed using Student’s t-test. The *P* values less than 0.05 were considered statistically significant. The sample sizes are indicated in the figure legends.

## Results

### Temsirolimus induces progerin clearance and ameliorates nuclear shape in HGPS cells

As rapamycin promotes the clearance of progerin via autophagy [26], we tested the ability of the rapamycin analog temsirolimus to ameliorate the HGPS cellular phenotype. Through cytotoxicity assays, we determined the non-toxic concentration of temsirolimus in HGPS and control fibroblast cultures (S1 Fig) and was found that 1 μM temsirolimus produced toxicity levels that were similar to mock-treated cells; this concentration was subsequently used in all experiments. Cells were treated daily with fresh medium supplemented with 1μM temsirolimus or vehicle (DMSO).

**Fig 1:**
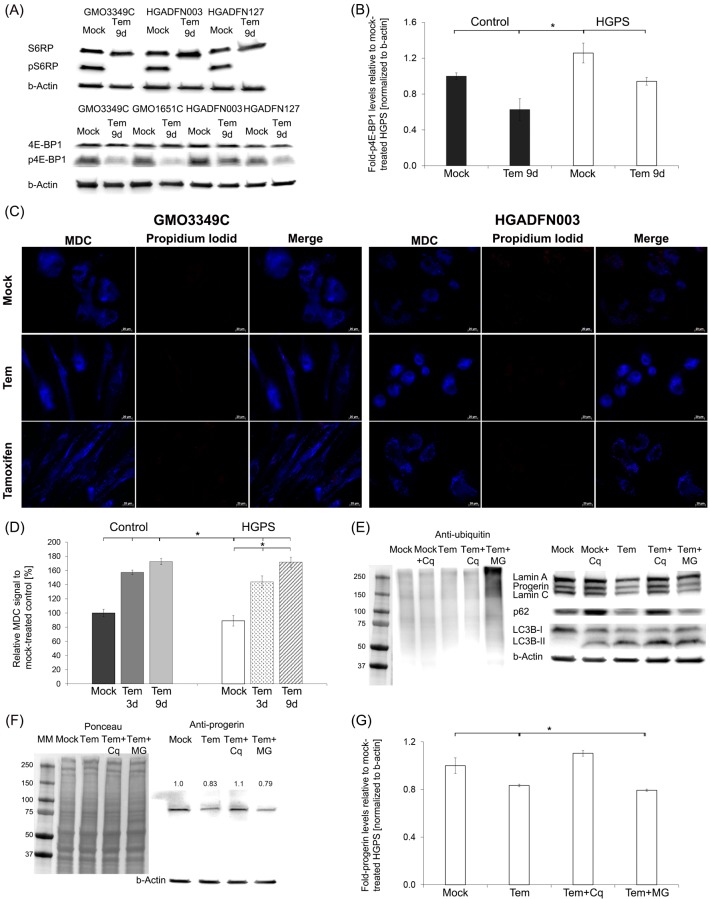
Temsirolimus increases progerin clearance via autophagy. (A) Representative Western blot of control (GMO3349C) and HGPS (HGADFN 003, HGADFN127) fibroblasts that were either mock-treated or Temsirolimus-treated (Tem) for a period of 9 days. Antibodies directed against the indicated proteins were used (S6RP, pS6RP, 4EBP1, and p4EBP1). (B) Quantification of p4EBP1 in control (GMO3349C, GMO1651C) and HGPS (HGADFN003, HGADFN127) cells treated with either the vehicle or 1 μM Temsirolimus for 9 days (n = 3) normalized to β-Actin. Levels are presented as the fold-expression relative to control cells. (C) Immunofluorescence staining of autophagosomes with the same cells as in (A). MDC (monodansylcadaverin) stained autophagosomes whereas propidium iodide was used as a marker for cell death. Tamoxifen was included as a positive control. (D) The same cells and culture conditions as in (A) were used to measure autophagy as described in Methods. Data are expressed as the mean ± S.D. (*p-value ≤ 0.05; n = 5). (E) Representative western blot of HGPS lysates. Cells were mock-treated or treated with 1.0 μM Temsirolimus, or 1.0 μM Temsirolimus with MG132 (MG) or chloroquine (Cq). Left panel: Western blot was probed for ubiquitin. Right panel: Western blot probed with antibodies specific for the indicated proteins lamin A/C, p62, LC-3B-I and II, and β-actin (n = 3). (F) Representative western blot using the same cells and culture conditions as described in (E). Left panel corresponds to Ponceau-S red staining of the western blot. Right panel: Western blot was probed with antibodies specific for the indicated proteins progerin, and β-actin (n = 3). Numbers above progerin bands represent the fold-expression. (G) The fold-expression for progerin was determined in each sample analyzed by western blotting with the anti-progerin antibody in panel (F) and was normalized to β-Actin. Levels are presented as the fold-expression relative to mock-treated control cells (*p-value ≤ 0.05; n = 5).

To examine whether temsirolimus inhibits the mTOR-signaling pathway as shown for rapamycin [Cao et al., 2011], we analyzed the phosphorylation status of the down-stream targets 4E-BP1 and S6RP (Fig 1A and 1B). As expected, temsirolimus lowered the phosphorylated 4E-BP1 and S6RP protein levels, indicating that the mTOR-signaling pathway was inhibited. Consequently, temsirolimus treatment efficiently stimulated autophagy as indicated by the monodansylcadaverine (MDC) signal on labeled autophagosomes **(**Fig 1C). Co-staining with propidium iodide, a marker of cell death, revealed that temsirolimus exerted no cytotoxic effects after 9 days of treatment in control and HGPS cells. Temsirolimus treatment induced similar results compared to those observed with tamoxifen, which served as a positive control for autophagy induction (Fig 1D). Increased MDC levels were observed in control (1.57-fold, p = 0.016) and HGPS (1.44-fold, p = 0.023) fibroblasts by day 3, and further increased by day 9 (Control = 1.72-fold, p = 0.001; HGPS = 1.71-fold, p = 0.014) (Fig 1D). Thus, temsirolimus significantly stimulated autophagy in both normal and HGPS fibroblasts (Fig 1D). To validate further the impact of temsirolimus on autophagy, HGPS fibroblasts were treated with temsirolimus for 8 days and then exposed to temsirolimus in combination with 25 μM chloroquine diphosphate (Cq), an inhibitor of autophagy, or 1 μM MG132, a proteasome inhibitor, for 12h (Fig 1E, 1F and 1G). Temsirolimus-treated HGPS cells exposed to MG132 demonstrated the accumulation of ubiquitinated proteins (Fig 1E), whereas those exposed to chloroquine exhibited a significant increase in LC3B-II levels, indicating that autophagosomes accumulated when autophagy was blocked (Fig 1E) [36]. LC3B-II levels were also increased in HGPS cells treated with temsirolimus alone (Fig 1E). LC3B-II accumulated even more in cells treated with both temsirolimus and chloriquine (Fig 1E). Autophagosomes (LC3-II) accumulation occurred in both case when autophagy was stimulated by temsirolimus or inhibited by chloriquine. We assessed the autophagic flux by determining the levels of p62, a protein that binds to LC3B-II and ubiquitinated proteins and is degraded in the autophagolysosomes. Cells treated with chloriquine exhibited increased p62 levels indicating that autophagic degradation was inhibited (Fig 1E). In contrast, p62 levels were reduced in cells treated with temsirolimus, indicating that autophagy flux was increased (Fig 1E). Moreover, progerin levels were reduced by 17% (p = 0.006) in temsirolimus-treated HGPS cells and even further reduced in temsirolimus-MG132-treated cells (21%, p = 0.002) compared with mock-treated cells (Fig 1F and 1G). Collectively, these data indicate that temsirolimus activates autophagy and thereby enhances progerin clearance in HGPS cells.

**Fig 2:**
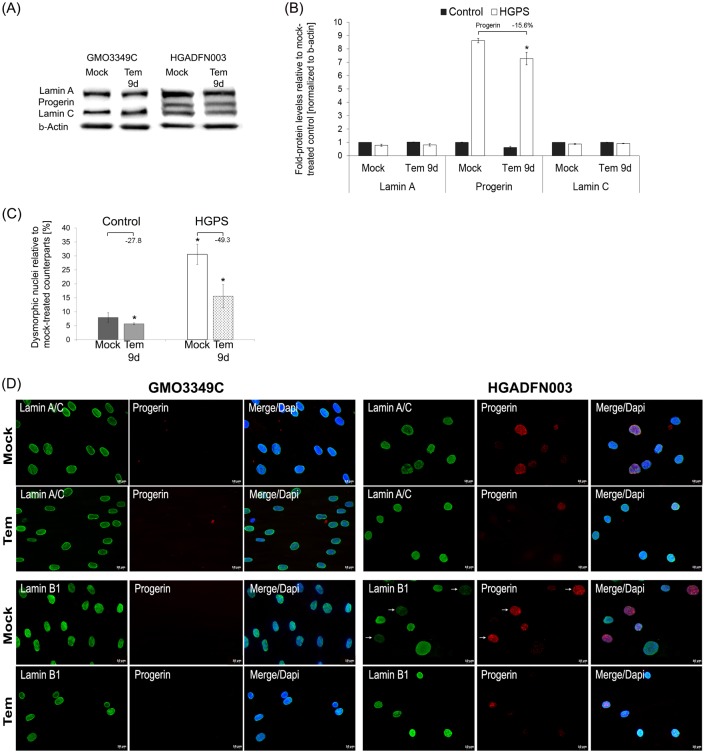
Temsirolimus ameliorates lamin status and nuclear shape in HGPS cells. (A) Representative Western blots of lamin A/C, progerin, and β-actin in control and HGPS total cell extracts isolated from either mock-treated cells or cells treated with 1.0 μM Temsirolimus daily for a period of 9 days. (B) Fold-expression of lamin A, progerin, and lamin C was determined for each sample analyzed by western blotting with anti-lamin A/C antibody in panel (A) and normalized to βan -Actin (*p-value ≤ 0.05; n = 5). (C) The frequency of misshapen nuclei (dysmorphic) after 9 days of treatment with either the vehicle or 1.0 μM Temsirolimus. An average of 900 nuclei were examined for each condition, and each experiment was repeated 3 times. (D) Immunochemistry was performed on mock-treated or Temsirolimus-treated control (GMO3349C) and HGPS (HGADFN003) fibroblasts after 9 days using antibodies against the indicated proteins (lamin A/C, progerin, and lamin B1). Representative images are shown (n = 4). Scale-bar: 20 μm.

Next, we performed Western blot analyses and quantified the levels of A-type lamins in cells treated daily for 9 days with the vehicle or temsirolimus (Fig 2A and 2B). In accordance with previous studies using rapamycin [26, 37], temsirolimus treatment reduced progerin levels by an average of 16% and induced no change in the levels of lamin A and lamin C (Fig 2B). Collectively, temsirolimus improved A-type lamin status in HGPS cells by increasing progerin clearance. For comparison, we also tested the effect of another FDA approved-rapalog, everolimus, and found that at similar concentrations, everolimus induced comparable positive effects on HGPS cultures regarding cell growth, autophagy and progerin clearance (S2 Fig).

HGPS nuclei are highly dysmorphic and exhibit numerous alterations, including nuclear envelope abnormalities and reduced levels of lamin B1 [4, 38]. Nine days of temsirolimus treatment significantly reduced the number of dysmorphic nuclei in HGPS cultures (Fig 2C). Additionally, in mock-treated cultures, most HGPS nuclei exhibited increased progerin accumulation at the nuclear envelope, with aggregates in certain areas as revealed when stained with anti-progerin antibodies (Fig 2D, upper panels). The HGPS nuclei that appeared normal showed reduced progerin staining [20]. Overall, both the number of bright progerin-positive nuclei and the signal intensity of these nuclei were reduced in temsirolimus-treated HGPS cells compared with mock-treated HGPS cells. Furthermore, the lamin B1 signal was barely detectable in untreated HGPS nuclei that were strongly labeled for progerin (Fig 2D, lower panels), whereas the signal intensity of lamin B1 was increased concomitantly with the reduced progerin signal in temsirolimus-treated HGPS cells, (Fig 2D). Collectively, temsirolimus treatment ameliorates HGPS nuclear morphology by reducing progerin levels.

### Long-term temsirolimus treatment improved cell growth and progerin clearance via autophagy in HGPS cells

**Fig 3:**
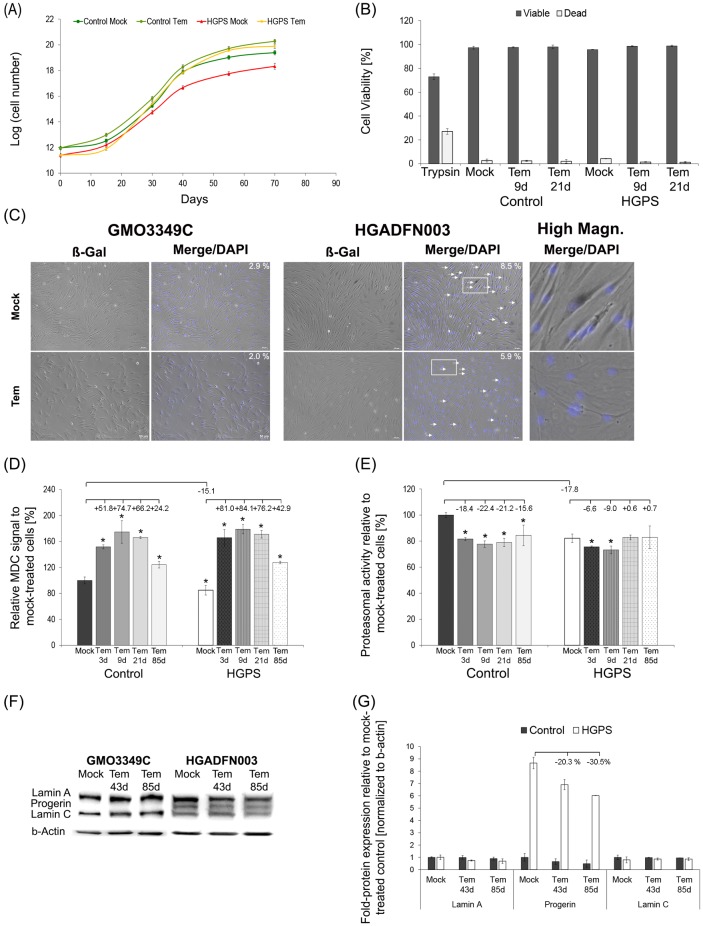
Temsirolimus further enhances progerin clearance during long-term treatment. (A) Cumulative population doublings were calculated as stated in Methods for control and HGPS fibroblasts at each indicated day. Cells were either mock-treated (vehicle DMSO) or treated with 1.0 μM Temsirolimus. (B) Viability assay for control and HGPS cells either mock-treated or treated with temsirolimus for 9 and 21 days. The assay was performed as stated in Materials and Methods. Trypsin was added as a positive control. Data are presented as the mean ± S.D. (*p-value ≤ 0.05; n = 4). (C) Mock-treated and temsirolimus-treated control and HGPS fibroblasts were used to detect senescence by b-galactosidase staining. Cells were cultured and treated with either the vehicle or temsirolimus for 55 days before they were stained with b-Galactosidase (b-Gal). Numbers indicate the frequency of b-Gal senescent cells. Right panel corresponds to high magnification images of b-Gal-positive HGPS fibroblasts that were mock-treated and temsirolimus-treated. Scale bar: 50 μM (n = 3). (D) The same cells and culture conditions as described in (A) were used to measure autophagy with monodansylcadaverine (MDC). Data are expressed as the mean ± S.D. (*p-value ≤ 0.05; n = 4). (E) Proteasomal activity was determined in the same cells used in (A) by measuring chymotrypsin-like proteasome activity. Cells were mock-treated or treated with 1.0 μM Temsirolimus every other day for the indicated period. The percentage of activity was calculated relative to mock-treated cells. Data are expressed as the mean ± S.D. (*p-value ≤ 0.05; n = 5). (F) Representative western blots of A-type lamins in control and HGPS fibroblasts. Cells were mock-treated or treated every other day with 1.0 μM Temsirolimus for the indicated period. The blots were probed with anti-lamin A/C and anti-β-actin antibodies (n = 3). (G) Fold-expression of A-type lamins were determined in each sample analyzed by western blotting with anti-lamin A/C antibody in panel (H). Levels are presented as the fold-expression ± S.D. relative to mock-treated normal cells (n = 3).

To test the efficiency of temsirolimus during long-term treatment, cells were treated every other day with 1 μM Temsirolimus or vehicle and passaged several times before analysis at the indicated time points (Fig 3).

Mock- and temsirolimus-treated normal fibroblasts exhibited a sustained growth rate during throughout the duration of 85 days (Fig 3A). Conversely, the mock-treated HGPS fibroblasts reached a growth plateau after a certain passage number, indicating the approach to cellular senescence. After a period of slow growth, temsirolimus-treated cells exhibited higher growth rates than mock-treated counterparts and for a longer period than 70 days (Fig 3A) [39]. Consistent with these findings, viability assays confirmed that temsirolimus increased the number of viable cells in HGPS cultures (Fig 3B). By day 9 of treatment, the number of viable cells was higher compared with mock-treated HGPS cultures. The number of viable HGPS cells was even further increased by day 21 of temsirolimus treatment (Fig 3B). To determine whether temsirolimus treatment could delay entry into cellular senescence, we quantified the number of cells exhibiting senescence-associated β-galactosidase positive signals at day 55 of temsirolimus treatment (Fig 3C). In the absence of temsirolimus, the percentage of senescent cells was higher in HGPS (8.5%) than in control (2.9%) cultures (Fig 3C). In the presence of temsirolimus, the number of ß-Gal-positive cells was decreased in both control (2.0%) and HGPS (5.9%) cultures (Fig 3C). To maintain proteostasis, protein turnover is regulated by autophagy and the ubiquitin-proteasome system (UPS) [40]. We determined autophagy and proteasome activity levels in fibroblast cultures treated with temsirolimus for long-term periods of time. Profiling autophagy activity at different time points indicated that temsirolimus induced, a maximum stimulation at day 9, with levels slowly decreasing by day 21 and decreasing even further by day 85 (Fig 3D). Regardless, autophagy remained significantly higher than in mock-treated counterparts (Fig 3D). Analysis of proteasome activity showed that temsirolimus further decreased its activity in control and HGPS cultures (Fig 3E). This result was in accordance with a previous study indicating that rapamycin and its analogs inhibit proteasome activity [41]. Simultaneously with the reduction in autophagy levels at day 21 of temsirolimus treatment, proteasome activity showed an increasing trend in both normal and HGPS cells, suggesting that activation of an adaptation response mechanism occurred during long-term treatment (Fig 3D and 3E). Western blot analyses of long-term cultures showed reduced levels of progerin in HGPS cells treated for 85 days (Fig 3F and 3G). Analysis of the levels of A-type lamins indicated that progerin was reduced by an average of 30.5% (p = 0.004) at day 85 (Fig 3G). Collectively, these findings indicate that long-term temsirolimus treatment further enhances progerin clearance and delays senescence in HGPS cells.

### Effect of temsirolimus on mitochondrial function in HGPS cells

Mitochondrial dysfunction has been identified as a hallmark of accelerated aging [42]. This earlier study demonstrated that proteins involved in glycolysis were increased whereas proteins involved in oxidative phosphorylation were decreased in HGPS [42]. We therefore investigated the effect of temsirolimus on mitochondrial function in control and HGPS fibroblasts. To date, this is the first study analyzing the effect of a rapalog on mitochondrial dysfunction.

**Fig 4:**
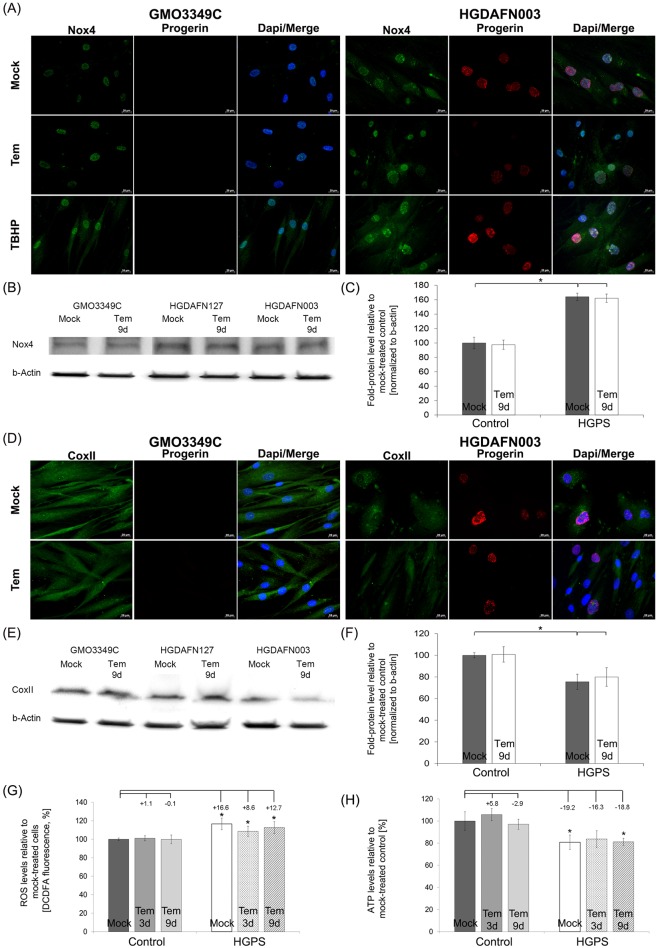
Temsirolimus does not impact HGPS mitochondrial dysfunction. (A) Immunohistochemistry was performed on mock-treated or temsirolimus-treated control (GMO3349C) and HGPS (HGADFN003) fibroblasts (9 days of treatment). Antibodies against NADPH oxidase subunit 4 (Nox4) and progerin were used. Representative images are shown (n = 4). Scale-bar: 20 μm. (B) Western blot analysis of control (GMO3349C) and HGPS cells (HGADFN003, HGADFN127) treated with 1 μM temsirolimus for 9 days were used. Antibodies against Nox4 and β-actin were used. A representative image is shown (n = 4). (C) Quantification of Nox4 levels normalized to β-actin and presented as the percentage relative to control cells (*p-value ≤ 0.05; n = 4). (D) Immunochemistry was performed on mock-treated or temsirolimus-treated control (GMO3349C) and HGPS (HGADFN003) fibroblasts at day 9. Antibodies against cytochrome c oxidase subunit II (CoxII) and progerin were used. Representative images are shown (n = 4). Scale-bar: 20 μm. (E) Western blot analysis of control (GMO3349C) and HGPS cells (HGADFN003, HGADFN127) treated with 1 μM Temsirolimus for 9 days were used. Antibodies against CoxII, and β-actin were used. Representative image is shown (n = 4). (F) Quantification of CoxII levels normalized to β-actin and presented as the percentage relative to control cells (*p-value ≤ 0.05; n = 4). (G) Intracellular ROS levels were determined by measuring oxidized dichlorofluorescein (DCF) levels, as described in Methods. Data represent the mean percentage ± S.D. (*p-value ≤ 0.05; n = 5) relative to mock-treated counterparts. (H) Cellular ATP levels were measured using a CellTiter Glo assay, as described in Methods. Data represent the mean percentage ± S.D. (*p-value ≤ 0.05; n = 5) relative to mock-treated counterparts.

We examined whether temsirolimus could ameliorate the activity of NADPH oxidase whose function is to produce reactive oxygen species (ROS) (Fig 4A). Nox4 is one of the isoforms of NADPH oxidases, which are enzymes that transport electrons across the plasma membrane and generate superoxide and reactive oxygen species (ROS) [43]. Mock-treated HGPS fibroblasts exhibited increased staining of Nox4 in the nucleus and cytoplasm (Fig 4A). In contrast, mock-treated control cells showed weak staining throughout the nucleus. Temsirolimus did not affect the levels or the distribution of Nox4 in control and HGPS cells (Fig 4A). These data were further confirmed by Western blot analyses (Fig 4B and 4C). The finding that Nox4 was increased in HGPS cells suggested that high levels of superoxide and reactive oxygen species were produced.

Previous studies have shown decreased levels of cytochrome c oxidase subunit II (CoxII) in HGPS cells [44, 45]. To further validate this finding, we determined the levels of CoxII an essential component of the mitochondrial respiratory chain [46, 47] (Fig 4B). In accordance with these studies, Western blot analyses indicated that CoxII levels were indeed reduced in HGPS cells compared with normal cells (Fig 4D–4F). Immunohistochemistry showed that CoxII cytoplasmic aggregates were present in bright progerin-positive HGPS cells (Fig 4D); however, overall CoxII signals were significantly reduced in HGPS cells relative to normal cells (Fig 4D). In HGPS cells treated with temsirolimus, CoxII signals remained weak (Fig 4D). These data were further confirmed by Western blot analysis (Fig 4E and 4F). Collectively, these data support the conclusion that temsirolimus treatment of HGPS cells does not improve mitochondrial function.

To further validate these findings, we evaluated the effect of temsirolimus by quantifying ROS and ATP levels in HGPS cells. Mock-treated HGPS fibroblasts exhibited a significant increase in basal ROS levels (16.6%, p = 0.003) compared with normal cells (Fig 4G) [20]. Temsirolimus treatment of normal and HGPS cells resulted in no significant changes in ROS levels (Fig 4G). The basal levels of ATP in HGPS fibroblasts were significantly reduced (19.2%, p = 0.032) compared with mock-treated normal cells (Fig 4H) [20]. Temsirolimus treatment induced no changes in the ATP levels in either normal or HGPS fibroblasts (Fig 4H). To further validate these findings, we tested the effect of another rapalog, everolimus and found that similarly to temsirolimus, everolimus neither reduce ROS levels nor increased ATP levels in treated HGPS cells (S2 Fig).

Collectively, our findings indicate that the levels of Nox4, CoxII, and ATP were reduced in HGPS cells, whereas ROS levels were increased. In addition, none of these parameters were improved after temsirolimus treatment.

Next, oxygen consumption, which is undertaken by mitochondria to produce ATP through oxidative phosphorylation, was measured in HGPS cells; glycolysis was also quantified by measuring the levels of extracellular lactate using a dual assay kit as described in the Methods (S2A Fig). Basal oxygen consumption was reduced in HGPS cells relative to normal cells (S2A Fig); the former showed a further reduction in oxygen consumption in the presence of temsirolimus (S2A Fig). In addition, the basal rate of glycolysis in HGPS cells was increased relative to normal cells, as reported previously [45]. Temsirolimus treatment reduced glycolysis in both control and HGPS cells as indicated by lower levels of lactate, the end product of glycolysis (S2A Fig).

To further investigate the effect of temsirolimus on mitochondrial functions cells were grown in either glucose or galactose medium supplemented with the vehicle or temsirolimus for up to 72 hours (S2B–S2D Fig). Cells grown under galactose conditions are forced to rely on mitochondrial oxidative phosphorylation for ATP generation and consequently are more sensitive to mitochondrial perturbation than cells grown in glucose media [48, 49]. Cells with mitochondrial deficiency grow slower than normal cells because they are unable to adapt to galactose media [45]. In our study, normal cells were able to adapt to galactose media and showed sustained growth after temsirolimus treatment for a period of 48 hours (S2B Fig). In contrast, temsirolimus treated-HGPS cells showed reduced growth rates after 24 hours in the presence of galactose media and then increasing to mock-treated levels after 48 hours (S2B Fig). Analysis of mitochondrial function showed no differences in ROS and ATP levels in control cells under galactose medium for a period of 3 days (S2D and S2E Fig). HGPS cells grown in galactose medium for 3 days showed increased ROS levels and decreased ATP levels (S2C and S2D Fig). In the presence of glucose and galactose, temsirolimus treatment induced no further changes in ROS and ATP levels in control and HGPS fibroblasts (S2C and S2D Fig). Together, the different parameters by which we assessed mitochondrial oxidative phosphorylation in HGPS cells indicate that temsirolimus does not rescue this function.

### Temsirolimus does not reduce ROS and superoxide production in HGPS cells

**Fig 5:**
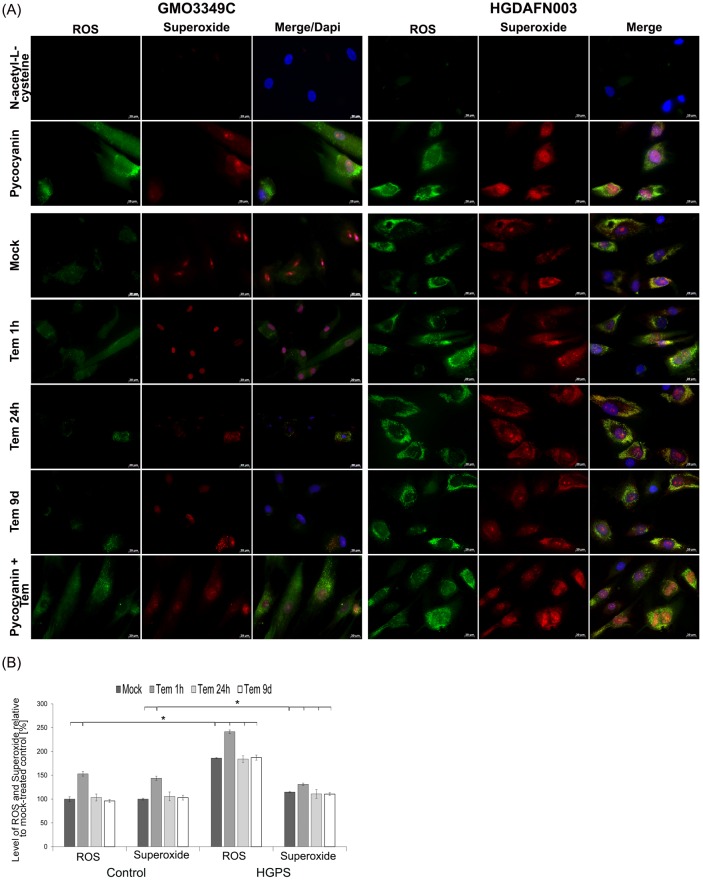
Temsirolimus does not impact the levels of reactive oxygen species and superoxide in HGPS cells. (A) Immunochemistry was performed on control (GMO3349C) and HGPS (HGADFN003) fibroblasts mock-treated or temsirolimus-treated for the indicated periods. Live cells were stained with an oxidative stress detection reagent for ROS and a superoxide detection reagent. The negative control was treated with N-acetyl-L-cysteine to quench the signal intensity. The positive control was treated with pycocyanin to induce ROS production. DNA was stained with Hoechst 33342. Representative images are shown (n = 4). Scale-bar: 20 μm. (B) The same cells and detection reagents as in (A) were used to perform a 96-well microplate assay as described in Methods. The percentage of activity was calculated relative to mock-treated control cells. Data are expressed as the mean ± S.D. (*p-value ≤ 0.05; n = 4).

As temsirolimus did not improve the levels of the endogenous source of cellular ROS (Nox4), we further evaluated ROS and superoxide levels by immunohistochemistry (Fig 5A and 5B). Strong ROS and superoxide signals were detected in HGPS cells compared with mock-treated normal cells (Fig 5A). Indeed, normal cells exhibited weak staining for ROS in the cytoplasm and for superoxide in the nucleus, whereas HGPS cells were brightly labeled and exhibited clusters of ROS signals close to the outer nuclear membrane as well as superoxide clusters throughout the cell (Fig 5A). Treatment with temsirolimus induced no changes in the distribution of ROS and superoxide signals in both normal and HGPS fibroblasts in comparison with mock-treated counterparts (Fig 5A).

Furthermore, treatment with pycocyanin, a compound that directly oxidizes intracellular pools of NAD(P)H and glutathione (GSH) [50], caused the accumulation of ROS and superoxide in both control and HGPS cells (Fig 5A, (upper panel). Co-treatment with temsirolimus did not prevent the accumulation of ROS and superoxide induced by pycocyanin in both control and HGPS cells (Fig 5A, last panel).

Using a dual kit assay for the measurement of ROS and superoxide (see Methods), we found that temsirolimus treatment for 1 hour induced a significant increase in ROS and superoxide in both normal and HGPS fibroblasts (Fig 5B). The levels of ROS and superoxide returned to those of mock-treated cells after 24 hours of temsirolimus treatment and remained at similar levels after 9 days. These results further demonstrate that within the first hours of exposure, temsirolimus exerts some degree of mitochondrial toxicity that appears to dissipate under long-term treatment, most likely due to the cell’s adaptive response mechanisms.

### Temsirolimus slightly improves DNA damage in HGPS cells

**Fig 6:**
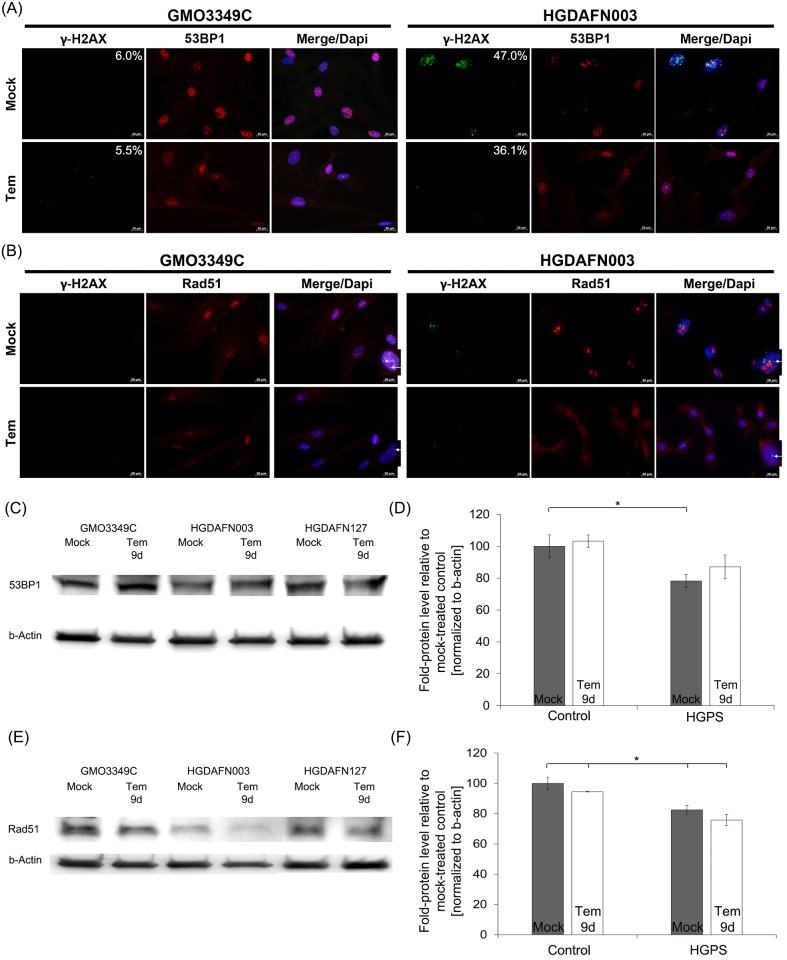
Temsirolimus does not impact DNA damage levels in HGPS cells. (A) Immunohistochemistry of control (GMO3349C) and HGPS (HGPSFN003) cells either mock-treated or treated with 1.0 μM temsirolimus for a period of 9 days. Antibodies against γH2A.X and 53BP1 were used (n = 4). Scale bar: 20 μm. (B) Immunohistochemistry of control (GMO3349C) and HGPS (HGPSFN003) cells either mock-treated or treated with 1.0 μM temsirolimus for a period of 9 days. Antibodies against γH2A.X and Rad51 were used (n = 4). Higher magnification pictures on the right side were added and white arrows indicate localization of γH2A.X and Rad51. Scale bar: 20 μm. (C) Western blot analysis of control (GMO3349C) and HGPS cells (HGADFN003, HGADFN127) treated with 1 μM Temsirolimus for 9 days were used. Antibodies against 53BP1 and β-actin were used. Representative image is shown (n = 4). (D) Quantification of 53BP1 levels normalized to β-actin and presented as the percentage relative to control cells (*p-value ≤ 0.05; n = 4). (E) Western blot analysis of control (GMO3349C) and HGPS cells (HGADFN003, HGADFN127) treated with 1 μM temsirolimus for 9 days were used. Antibodies against Rad51 and β-actin were used. Representative image is shown (n = 4). (F) Quantification of Rad51 levels normalized to β-actin and presented as the percentage relative to control cells (*p-value ≤ 0.05; n = 4).

Reactive oxygen species (ROS) and oxidizing agents are known to induce DNA damage [51]. Consistent with the increased levels of ROS in HGPS cells, the numbers of nuclei exhibiting DNA damage foci (γH2A.X foci) were significantly higher in HGPS (47%) compared with normal fibroblast cultures (6%) (Fig 6A). Temsirolimus treatment induced a decreased number of γH2A.X-positive HGPS nuclei (36.1%) signifying a reduction in DNA damage. We also assessed the repartitioning of two DNA damage repair factors, the 53BP1, which promote nonhomologous end-joining (NHEJ) [52], and Rad51, which is involved in homologous recombination (HR) [53] (Fig 6A and 6B). Co-staining of γH2A.X with 53BP1 showed colocalization of both proteins at sites of DNA damage foci, but their numbers were reduced in temsirolimus-treated HGPS cells (Fig 6A). 53BP1 protein levels were reduced in mock-treated HGPS cells but showed a tendency to increase in the presence of temsirolimus (Fig 6C). In contrast, co-staining of γH2A.X with Rad51 showed no co-localization at sites of γH2A.X foci in temsirolimus- or mock-treated HGPS cells (Fig 6B). In control nuclei Rad51 was distributed throughout the nucleoplasm and remained similarly distributed in the presence of temsirolimus (Fig 6B). In contrast, Rad51 formed large nuclear aggregates in mock-treated HGPS cells but became more diffused after temsirolimus treatment (Fig 6B). Thus, temsirolimus treatment caused a reduction in Rad51 protein levels in both normal and HGPS cells (Fig 6D).

Collectively, our results indicate that temsirolimus slightly lowered the amount of DNA damage in HGPS cells, which appeared to be facilitated by engaging the 53BP1 repair process [52].

## Discussion

In this study, we evaluate the ability of the rapamycin analog temsirolimus to reverse some of the hallmarks of the HGPS cellular phenotype [1]. Temsirolimus is a dihydroxymethyl propionic acid ester of rapamycin that exhibits improved solubility and specifically inhibits mTOR signaling with an effectiveness comparable to that of rapamycin [54]. Moreover, temsirolimus (Torisel, CCI-779) is approved by the Food and Drug Administration (FDA) for human use. The safety, tolerability and efficacy of temsirolimus have been evaluated in phase I, II, and III clinical trials (https://clinicaltrials.gov), which have indicated that temsirolimus is less immune-suppressive and exhibits improved pharmacological characteristics compared to rapamycin [55].

In recent years, rapamycin has emerged as a potential candidate drug for clinical trials of children with HGPS disease [26, 37, 56], and recently the Progeria Research Foundation (progeriaresearch.org) launched a phase I trial of everolimus in April 2016. To date, temsirolimus and everolimus are both FDA-approved rapalogs and have been used in numerous clinical trials. They both share similar properties, with the only difference that temsirolimus is formulated for intravenous injection and everolimus for oral administration.

In this study, we investigated the efficacy of the analog temsirolimus to determine the extent to which this compound can correct HGPS cellular defects. Our findings support previous studies indicating that inhibition of the mTORC1 signaling pathway by rapamycin leads to autophagy activation in HGPS cells [26, 57], and we also provide evidence that progerin is degraded via the autophagy pathway [26, 37, 56, 58]. Indeed, temsirolimus treatment reduces progerin nuclear accumulation and improves the growth rate and lifespan of HGPS cells *in vitro*, as previously reported for rapamycin [26]. Such a reduction in progerin also ameliorates the HGPS nuclear morphology, as indicated by decreased nuclear envelope abnormalities, including blebbing and invaginations [26, 37, 56, 58]. Our study additionally shows restoration of normal levels of lamin B1 with temsirolimus treatment. Collectively, these studies indicate that temsirolimus partially rescues lamin A/C and lamin B1 status via the reduction of progerin content and thereby normalizes the nuclear morphology in HGPS cells. However, other critical cellular defects of HGPS cells, such as mitochondrial dysfunction and high levels of DNA damage, were not rescued by temsirolimus treatment.

### Temsirolimus treatment ameliorates HGPS proteostasis

Autophagy and ubiquitin-proteasome pathways constitute the two major degradation routes for maintaining proteostasis [36]. In this study, we found that HGPS cells exhibit alterations in their protein degradation systems, with reduced autophagy and proteasome activity, as reported previously [20]. HGPS cells show a strong activation of autophagy after temsirolimus treatment, leading to reduced levels of progerin. Autophagy stimulation occurs through the inhibitory action of temsirolimus on the mTOR pathway, as evidenced by reduced levels of the phosphorylated form of its downstream effectors’ ribosomal proteins, S6 and E4BP1. These findings are in accordance with previous rapamycin studies [26, 37, 56, 58]. However, we showed that the effect of temsirolimus was similar to that of rapamycin and was due to mTOR inhibition, we found that autophagy stimulation by temsirolimus was higher during short-term treatment, with maximum induction observed at day 9. Long-term temsirolimus treatment resulted in higher levels of autophagy compared to the basal levels in mock-treated cells, but a tendency toward decline by day 21 and further reduction was observed at day 85 of treatment. These findings indicated that temsirolimus efficiently stimulated autophagy in both normal and HGPS fibroblasts but its amplitude of activation in cells decreased during long-term treatment.

Proteasome activity was reduced during short-term temsirolimus treatment in both normal and HGPS cells. Rapamycin was previously shown to allosterically inhibit the activity of the 20S proteasome [59]. Rapamycin induces a conformational shift of the 20S proteasome, which results in compromised gating of substrates. Rapalogs might directly compete with canonical ligands for the same binding grooves, which manifests in weak-to moderate inhibition of chymotrypsin-like activity Osmulski, 2013 #1404}.

Proteasome activity in HGPS cells slightly increased during long-term temsirolimus treatment, which was concomitant with a reduction in autophagy stimulation. The inverse correlation between the activities of these two degradation pathways suggests that treatment with temsirolimus for a prolonged period of time (weeks) might lead to the activation of a cellular adaptation response to prevent a further decrease in proteasome activity. Alternatively, cells might become less sensitive to the drug during long-term treatment. Further studies are needed to determine how cells maintain a balance between autophagy and proteasome activity during long-term treatment; evaluating the effects of intermittent treatments with rapamycin or temsirolimus on proteostasis is warranted.

### Temsirolimus treatment does not impact HGPS mitochondria dysfunction

Previous studies have demonstrated increased levels of ROS and decreased levels of ATP in HGPS cells [20, 60]. Recently, a SILAC study provided important insight into the mitochondrial dysfunction of HGPS cells [45], showing that down-regulation of several components of the mitochondrial ATPase complex and up-regulation of glycolytic enzymes are features of progeria cells [45]. Thus, in HGPS cells, ATP production appears to occur through glycolysis rather than oxidative phosphorylation [45].

In our study, we detected decreased ATP levels and increased superoxide and ROS levels in HGPS fibroblasts, as well as higher levels of NADPH oxidase subunit 4 and cytochrome c oxidase subunit II levels in comparison to normal cells. The oxygen consumption rate in HGPS cells was lower, whereas glycolysis was enhanced.

Temsirolimus treatment did not significantly impact mitochondrial function in HGPS cells, with no effects on ROS, ATP, superoxide, NADPH oxidase subunit 4 and cytochrome c oxidase subunit II levels. Notably, temsirolimus treatment worsened the mitochondrial function status within the first hours of treatment in both normal and HGPS cells, although continued treatment did return the levels of those parameters to those of their mock-treated counterparts. Moreover, changing the energy source from glucose to galactose showed that HGPS cells were more sensitive to mitochondrial stress than normal cells, as evidenced by a further reduction in ATP and increases in ROS in HGPS cells grown in galactose medium. In fact, temsirolimus treatment did not prevent these changes. Interestingly, the inhibition of mTOR by rapamycin in cancer cells was shown to enhance aerobic glycolysis and to decrease uncoupled mitochondrial respiration [61]. This previous observation might explain why ROS and ATP levels were not rescued by temsirolimus treatment in HGPS cells. Further studies are needed to elucidate the limitations of the effect of rapamycin on mitochondrial function, as was recently also suggested in skeletal muscles from mice treated with rapamycin [62].

### Temsirolimus treatment has a limited impact on HGPS DNA damage levels

HGPS cells accumulate DNA double-strand breaks (DSBs), and progerin is thought to affect the rate of DSB repair [63]. Treatment with rapamycin was found to improve DNA damage repair by enhancing the levels of 53BP1. Nonetheless, other studies have reported a reduction in the proliferation rate of rapamycin-treated HGPS cells, and no benefit to DNA damage repair in cells from progeria mice treated with rapamycin was found [10]. In our cell-based study, temsirolimus enhanced DNA damage repair, as indicated by the reduced number of DNA damage foci and enhanced expression of 53BP1 in HGPS nuclei. However, the overall number of cells exhibiting DNA damage was only slightly decreased in the presence of temsirolimus. The persistence of high levels of DNA damage might be attributed to the remaining increased levels of ROS in temsirolimus-treated HGPS cells. Recent studies have provided evidence that antioxidant compounds, such as the ROS scavenger N-acetyl cysteine (NAC), or treatment with sulforaphane, an activator of the Nrf2 signaling pathway, can efficiently reduce ROS and DNA damage in HGPS cells [20, 64].

In summary, the HGPS fibroblasts used in this study exhibit a highly altered phenotype with extensive changes to A-type lamins, accompanied by nuclear dysmorphy. The purpose of this investigation was to characterize and quantitate the extent to which temsirolimus, a rapamycin analog with an improved clinical toxicity profile, could improve the HGPS cellular phenotype. Our findings indicate that temsirolimus restores a normal morphology to HGPS fibroblasts, improves their growth potential and delays senescence. Temsirolimus activates autophagy and enhances progerin clearance while decreasing proteasome activity in HGPS cells. During long-term cultures, temsirolimus appears to elicit a cellular adaptation response that leads to a reduction in its capacity to stimulate autophagy and concomitantly induces an increase in proteasome activity. These findings suggest that discontinuous temsirolimus treatment should be considered and further investigated. Because temsirolimus had no impact on DNA damage or mitochondrial function, and given that these two functions are defective in HGPS cells, they therefore must be corrected to normalize the HGPS cellular phenotype. Our findings suggest that in addition to the use of rapamycin analogs, additional treatment with antioxidant compounds might be necessary to reverse the remaining defects in HGPS cells. Alternatively, the use of drugs that can simultaneously stimulate autophagy and reduce ROS levels should be considered as reported previously [20]. With the increasing number of candidate compounds for treating HGPS disease [1, 65], further therapeutic avenues will require the identification of appropriate drug combinations and treatment regimens to target the numerous phenotypic hallmarks that characterize HGPS cells.

## Supporting Information

S1 Fig Cytotoxicity of temsirolimus and analysis of treatment periods. (A) Control cells were incubated for 48 hours with increasing concentrations of temsirolimus, as indicated. Mock-treated cells were treated with the vehicle DMSO alone. The percentage of dead cells was determined using a Cell Tox Green Kit, as described in Methods. All values are presented as the mean ± S.D. (*p-value ≤ 0.05; n = 3) relative to the mock-treated control.
(TIF)

S2 Fig Comparison of everolimus and temsirolimus. (A) Control cells were incubated for 48 hours with increasing concentrations of everolimus (Eve), as indicated. Mock-treated cells were treated with the vehicle (DMSO) alone. Temsirolimus-treated control cells (Tem, 1 μM) served as a control. The percentage of dead cells was determined using a Cell Tox Green Kit, as described in the Methods. All values are presented as the mean ± S.D. (*p-value ≤ 0.05; n = 3) relative to the mock-treated control. (B) Control and HGPS cells were treated with increasing concentrations of everolimus for 48 h, as indicated. Mock-treated and temsirolimus-treated cells served as controls. Autophagosome levels were determined using an MDC-Kit as described in the Methods. Data are presented as the mean ± S.D. (*p-value ≤ 0.05; n = 3) relative to the mock-treated control. (C) The same cells as in (B) were used to determine the cellular growth under different concentrations of everolimus. Data are presented as the mean ± S.D. (*p-value ≤ 0.05; n = 3) relative to mock-treated counterparts. (D) Control and HGPS cells were treated for 9 days with 1 μM everolimus or 1 μM temsirolimus. Mock-treated cells were treated with the vehicle DMSO alone. Autophagosome levels were measured using an Autophagy/Cytotoxicity dual-staining kit as described in the Methods. Data are presented as the mean ± S.D. (*p-value ≤ 0.05; n = 3) relative to the mock-treated control. (E) The same cells as in (D) were used to determine intracellular ROS levels by measuring oxidized dichlorofluorescein (DCF), as described in the Methods. Data are presented as the mean ± S.D. (*p-value ≤ 0.05; n = 3) relative to mock-treated control. (F) Cellular ATP levels were measured in the same cells as in (D) by using a CellTiter Glo assay, as described in the Methods. Data are presented as the mean ± S.D. (*p-value ≤ 0.05; n = 3) relative to the mock-treated control. (G) Cell growth of the cells used in (D) was determined and is presented as the mean ± S.D. (*p-value ≤ 0.05; n = 3) relative to mock-treated counterparts. (H) Representative Western blot of A-type lamins in HGPS cells that were treated with vehicle, everolimus (1 μM) or temsirolimus (1 μM) for 9 days. Antibodies against lamin A/C and β-actin were used (n = 3). Progerin clearance of everolimus and temsirolimus after 9 days is indicated and presented as the percentage after normalization to β-actin and relative to the mock-treated control.
(TIF)

S3 Fig Effect of temsirolimus under galactose conditions. (A) Oxygen consumption (VO_2_) and glycolysis were measured using MitoXpress Solution and a tetrazolium substrate, respectively. Cells were mock-treated and temsirolimus-treated in glucose medium for the indicated time periods and stained according to the manufacturer’s instructions. Fluorescence was measured to detect oxygen consumption, and absorbance was measured to detect glycolysis levels. Data are expressed as the mean ± S.D. (*p-value ≤ 0.05; n = 4) relative to mock-treated control cells. Antimycin A, an inhibitor of the mitochondrial electron transport chain, served as a control. (B) Control cells were incubated for the indicated times with 1 μM Temsirolimus in either glucose or galactose medium. Mock-treated cells were treated with the vehicle DMSO alone. The percentage of cell growth was determined. All values are presented as the mean ± S.D. (*p-value ≤ 0.05; n = 3) relative to mock-treated counterparts. (C) Intracellular ROS levels were determined as described in Methods. Cells were cultured in high glucose or galactose medium with either vehicle or 1 μM temsirolimus for the indicated period of time. Data represent the mean percentage ± S.D. (*p-value ≤ 0.05; n = 6) relative to mock-treated control cells. (D) Cellular ATP levels were determined using a CellTiter Glo assay, as described in Methods. Cells were treated as described in (C). Data represent the mean percentage ± S.D. (*p-value ≤ 0.05; n = 6) relative to mock-treated control cells.
(TIF)
