# Supplementary figures and images for: Temsirolimus Partially Rescues the Hutchinson-Gilford Progeria Cellular Phenotype
Source: PLoS One. 2016 Dec 29;11(12):e0168988. doi: 10.1371/journal.pone.0168988 (PMC5199099; doi:10.1371/journal.pone.0168988)

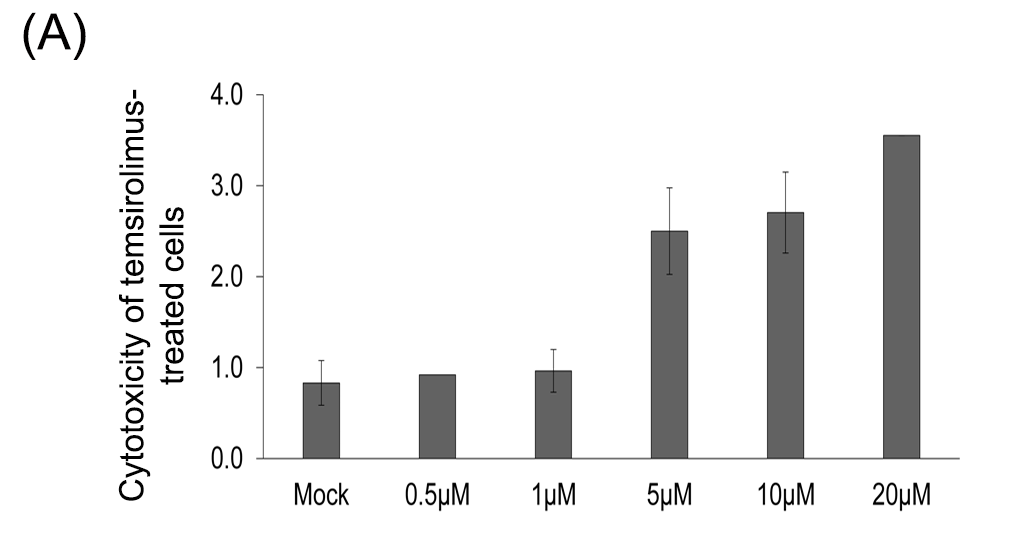

Supplement: S1 Fig — (A) Control cells were incubated for 48 hours with increasing concentrations of temsirolimus, as indicated. Mock-treated cells were treated with the vehicle DMSO alone. The percentage of dead cells was determined using a Cell Tox Green Kit, as described in Methods. All values are presented as the mean ± S.D. (*p-value ≤ 0.05; n = 3) relative to the mock-treated control. (TIF) [file pone.0168988.s001.tif]

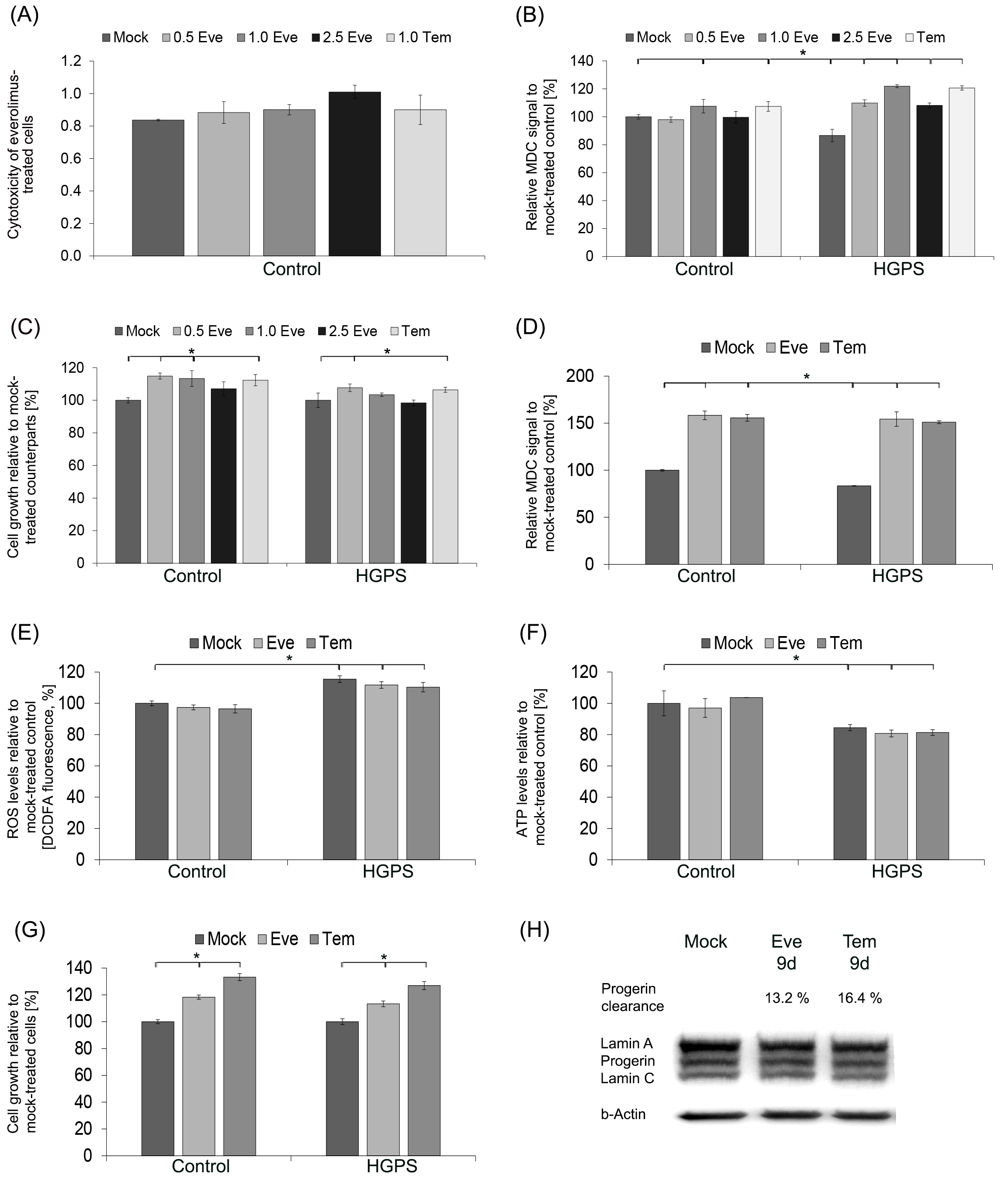

Supplement: S2 Fig — (A) Control cells were incubated for 48 hours with increasing concentrations of everolimus (Eve), as indicated. Mock-treated cells were treated with the vehicle (DMSO) alone. Temsirolimus-treated control cells (Tem, 1 μM) served as a control. The percentage of dead cells was determined using a Cell Tox Green Kit, as described in the Methods. All values are presented as the mean ± S.D. (*p-value ≤ 0.05; n = 3) relative to the mock-treated control. (B) Control and HGPS cells were treated with increasing concentrations of everolimus for 48 h, as indicated. Mock-treated and temsirolimus-treated cells served as controls. Autophagosome levels were determined using an MDC-Kit as described in the Methods. Data are presented as the mean ± S.D. (*p-value ≤ 0.05; n = 3) relative to the mock-treated control. (C) The same cells as in (B) were used to determine the cellular growth under different concentrations of everolimus. Data are presented as the mean ± S.D. (*p-value ≤ 0.05; n = 3) relative to mock-treated counterparts. (D) Control and HGPS cells were treated for 9 days with 1 μM everolimus or 1 μM temsirolimus. Mock-treated cells were treated with the vehicle DMSO alone. Autophagosome levels were measured using an Autophagy/Cytotoxicity dual-staining kit as described in the Methods. Data are presented as the mean ± S.D. (*p-value ≤ 0.05; n = 3) relative to the mock-treated control. (E) The same cells as in (D) were used to determine intracellular ROS levels by measuring oxidized dichlorofluorescein (DCF), as described in the Methods. Data are presented as the mean ± S.D. (*p-value ≤ 0.05; n = 3) relative to mock-treated control. (F) Cellular ATP levels were measured in the same cells as in (D) by using a CellTiter Glo assay, as described in the Methods. Data are presented as the mean ± S.D. (*p-value ≤ 0.05; n = 3) relative to the mock-treated control. (G) Cell growth of the cells used in (D) was determined and is presented as the mean ± S.D. (*p-value ≤ 0.05; n = 3) relat [file pone.0168988.s002.tif]

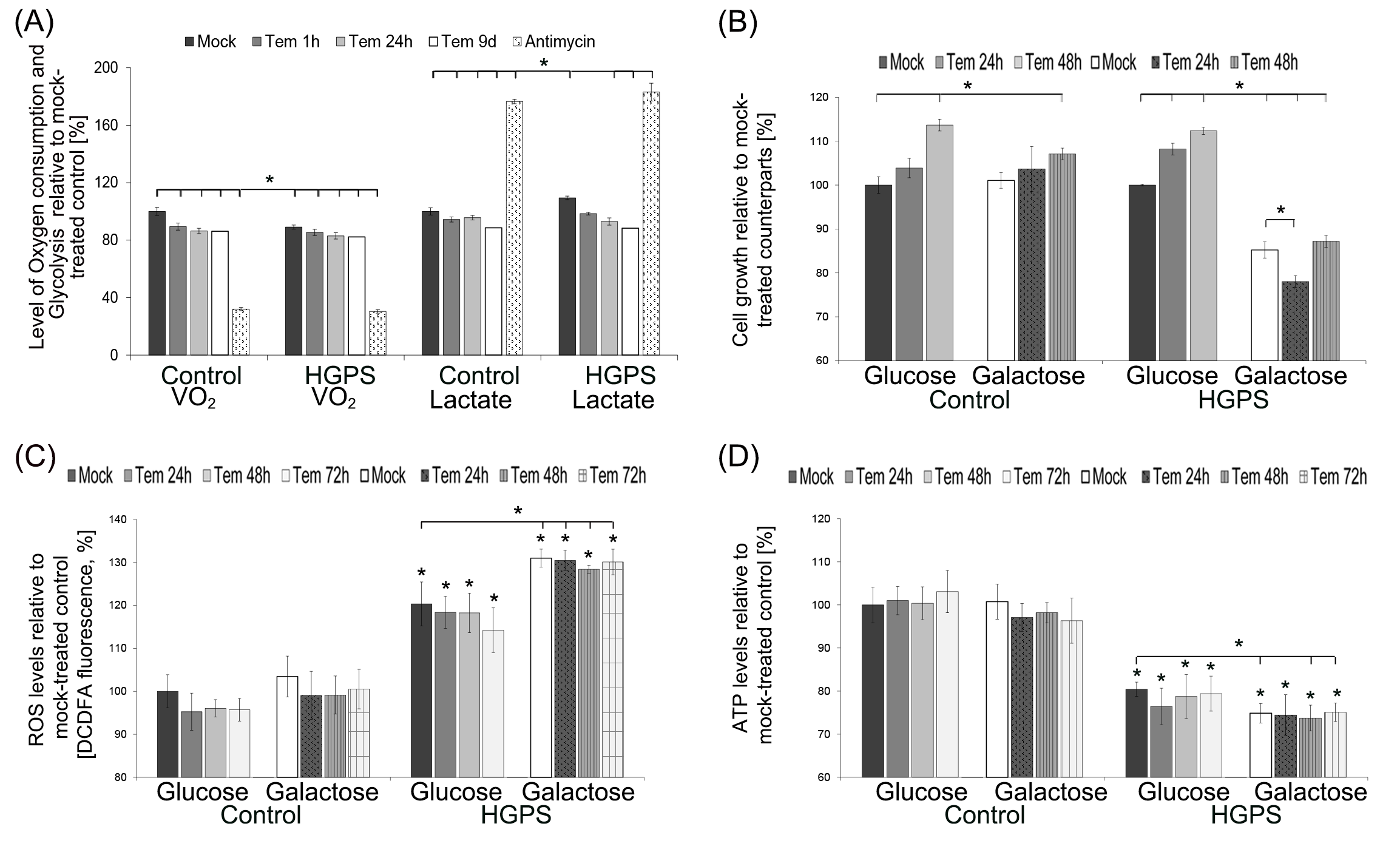

Supplement: S3 Fig — (A) Oxygen consumption (VO2) and glycolysis were measured using MitoXpress Solution and a tetrazolium substrate, respectively. Cells were mock-treated and temsirolimus-treated in glucose medium for the indicated time periods and stained according to the manufacturer’s instructions. Fluorescence was measured to detect oxygen consumption, and absorbance was measured to detect glycolysis levels. Data are expressed as the mean ± S.D. (*p-value ≤ 0.05; n = 4) relative to mock-treated control cells. Antimycin A, an inhibitor of the mitochondrial electron transport chain, served as a control. (B) Control cells were incubated for the indicated times with 1 μM Temsirolimus in either glucose or galactose medium. Mock-treated cells were treated with the vehicle DMSO alone. The percentage of cell growth was determined. All values are presented as the mean ± S.D. (*p-value ≤ 0.05; n = 3) relative to mock-treated counterparts. (C) Intracellular ROS levels were determined as described in Methods. Cells were cultured in high glucose or galactose medium with either vehicle or 1 μM temsirolimus for the indicated period of time. Data represent the mean percentage ± S.D. (*p-value ≤ 0.05; n = 6) relative to mock-treated control cells. (D) Cellular ATP levels were determined using a CellTiter Glo assay, as described in Methods. Cells were treated as described in (C). Data represent the mean percentage ± S.D. (*p-value ≤ 0.05; n = 6) relative to mock-treated control cells. (TIF) [file pone.0168988.s003.tif]
